# M. Orfila on the Absorption of Chemical Substances into the Blood, &c.

**Published:** 1842-11-19

**Authors:** 


*M. Orfila on the Absorption of Chemical Substances into the Blood, &c.* During the last two or three years, we have repeatedly called the attention of our readers to the very interesting researches of the distinguished toxicologist of Paris relative to the absorption of certain metallic salts, more especially those of arsenic, antimony and copper, into the animal system, and the means of detecting their existence in various organs of the body after death.

We learn from the following letter, addressed by him, during last April, to the Academy of Medicine, that he has been prosecuting his inquiries on this hitherto imperfectly examined point of pathological and physiological science; and we earnestly recommend all practical men not to neglect a diligent acquaintance with such subjects, as it is far from being improbable that the therapeutic action of many active medicines may become much better understood, and thereby the practice of physic be improved, by the discoveries of modern chemistry.

" The following results I have obtained," says M. Orfila, " from a great number of experiments, the details of which will shortly be published.

" 1. The diluted sulphuric, nitric, muriatic, and oxalic acids, are absorbed and may be detected in the urine.

" 2. The same acids, even in their concentrated condition, are also absorbed; but this probably occurs after their becoming blended with the watery juices of the stomach and bowels, the secretion of which is increased by the contact of such stimulating agents.

" 3. The absorption of the salts of lead, bismuth, tin, zinc, gold and silver, cannot be disputed, seeing that we find in the liver and also in the urine of animals, which have been poisoned with those substances, the metals which form their bases. All these metals are discoverable by the same analytic process; viz. carbonising the viscus with strong nitric acid, and treating the residue with aqua regia or with nitric acid.

" 4. The salts of mercury also are absorbed and carried by the blood into all the viscera of the body. To prove this, we have only to treat one of the viscera of an animal, which has been poisoned with a mercurial salt, with aqua regia, and to pass through the solution thus obtained a current of chlorine gas; or what is still better, to carbonate the viscera with a strong acid in a closed vessel, and to treat the residue with boiling aqua regia.

" 5. I have also detected in the viscera—more especially in the substance of the liver and in the urine—traces of the sulphuret of iodine, of nitrate of potash, of alum, of ammonia, and of sal ammoniac.

" The results now stated complete the suite of researches which I proposed to undertake on the subject of the absorption of poisonous matters derived from the mineral kingdom; they serve to establish beyond all doubt the correctness of the views which I explained in my former communications relative to the absorption of the salts of arsenic, antimony, and copper."—*Med. Chir. Rev.* Oct. 1842, *from Gaz. Medicale.*

